# Microplastics and dialysis: a new challenge for green nephrology

**DOI:** 10.1590/2175-8239-JBN-2026-0094en

**Published:** 2026-11-02

**Authors:** Rodrigo Bueno de Oliveira, Eric Aragão Corrêa, Peter Stenvinkel

**Affiliations:** 1 Universidade Estadual de Campinas, Faculdade de Ciências Médicas, Departamento de Clínica Médica, Campinas, SP, Brazil.; 2 Universidade Estadual de Campinas, Faculdade de Ciências Médicas, Laboratório para o Estudo Mineral e Ósseo em Nefrologia, Campinas, SP, Brazil.; 3 Karolinska University Hospital, Department of Renal Medicine, Stockholm, Sweden.

**Keywords:** Microplastics, Nanoplastics, Renal Insufficiency, Chronic, Dialysis, Renal Dialysis, Green Dialysis, Green Nephrology

## Abstract

Green nephrology has emerged as a pivotal strategy to mitigate the environmental impacts of kidney care. Within this framework, reducing plastic waste and lowering the carbon footprint are closely interconnected, as both aim to minimize unnecessary resource consumption and the environmental burden associated with healthcare delivery. The rapid growth of plastic production and use has led to widespread environmental pollution and the generation of enormous quantities of micro- and nanoplastics (MNPs) through plastic degradation. Increasing evidence suggests that exposure to MNPs may pose potential risks to human health. However, important questions remain unanswered regarding chronic kidney disease (CKD), dialysis therapies, and exposure to MNPs. In particular, the magnitude of exposure, the potential for accumulation in patients with impaired renal function, and the possible clinical consequences remain largely unknown. In this opinion paper, we discuss the emerging challenges and potential opportunities at the intersection of microplastics, dialysis, and green nephrology.

## INTRODUCTION

A single dialysis session can involve up to 8 kg of plastic materials and consume hundreds of liters of water^1,2,3^. With the exponential increase in the number of people suffering from CKD and the need for renal replacement therapy, the projected environmental impact associated with the delivery of dialysis care is enormous^4,5^. To reduce the ecological footprint related to kidney care is a real challenge^6^.

Over the past 15 years, strategies for delivering kidney care without harming the planet have been discussed, including efficient resource management, circular economy practices, prevention of CKD and its complications, and patient and community engagement. These strategies are collectively referred to as “green nephrology”^1,6,7,8^.

In addition to these environmental issues associated with kidney care, in recent years, evidence has linked the release of microparticles (< 5 mm) and nanoparticles (< 1 µm) of plastic materials (MNPs) to deleterious biological effects on eukaryotic cells and mammalian animals, including humans^9,10,11^.

Although the effects of MNPs on the pathogenesis of human diseases are still largely unknown, scientific documents have reported their presence in virtually all organs of the human body^12,13,14,15^. It is now known that MNPs can accumulate in kidney tissue and be excreted in urine, and experimental studies suggest that they may induce adverse biological effects in the kidney^10,11,12^.

It is plausible to speculate that with the progression of kidney failure, patients may experience a decrease in their ability to eliminate MNPs through urine, even though scientific confirmation on this aspect is still pending. Limited evidence suggests that patients with CKD may be exposed to various sources of plastics. Accordingly, two studies detected MNPs in hemodialysis and peritoneal dialysis fluids; another study based on mathematical modeling recommends that hemodialysis may release significant amounts of MNPs directly into the bloodstream^16,17,18^.

In this perspective/opinion article, the available evidence on MNPs and kidney care is discussed and integrated within the context of green nephrology. Furthermore, we discuss concerns, challenges, and opportunities surrounding dialysis care within the principles of green nephrology, with particular attention to this new environmental contaminant: MNPs.

## HOW DO CKD AND DIALYSIS BURDEN RELATE TO CLIMATE CHANGE?

CKD affects 14.2% of the global adult population, making it one of the most prevalent and fastest-growing non-communicable diseases (NCDs) worldwide^19^. There are 788 million people with kidney disease globally, of whom 3.6 million are on kidney replacement therapy (KRT), and 1.5 million people die annually from CKD. Worldwide, the prevalence of CKD varies substantially across regions and populations, with estimates ranging from around 5–20% depending on country and risk factors such as diabetes, hypertension, and socioeconomic conditions; for example, much higher rates are reported in Northern Africa, the Middle East, South Asia, and Central America compared with high-income regions^4^. This alarming trend contributed to the decision by the World Health Organization (WHO) to recognize CKD as an NCD of increasing global priority^4,20^.

Rising average global temperatures due to climate change have the potential to exacerbate the CKD burden, as they are linked with significant health hazards that affect kidney health^21,22^. In an era of accelerating climate change and ecological disruption, the kidneys may serve as the canaries in the coal mine of public health: highly sensitive, biologically responsive organs that register hemodynamic, osmotic, inflammatory, and toxic strain long before broader patterns of disease fully emerge.

This cycle closes as providing kidney care requires substantial environmental resources and generates large amounts of plastic waste, and, consequently MNPs, causing an environmental impact (Figure 1)^1,3,8^.

**Figure 1:**
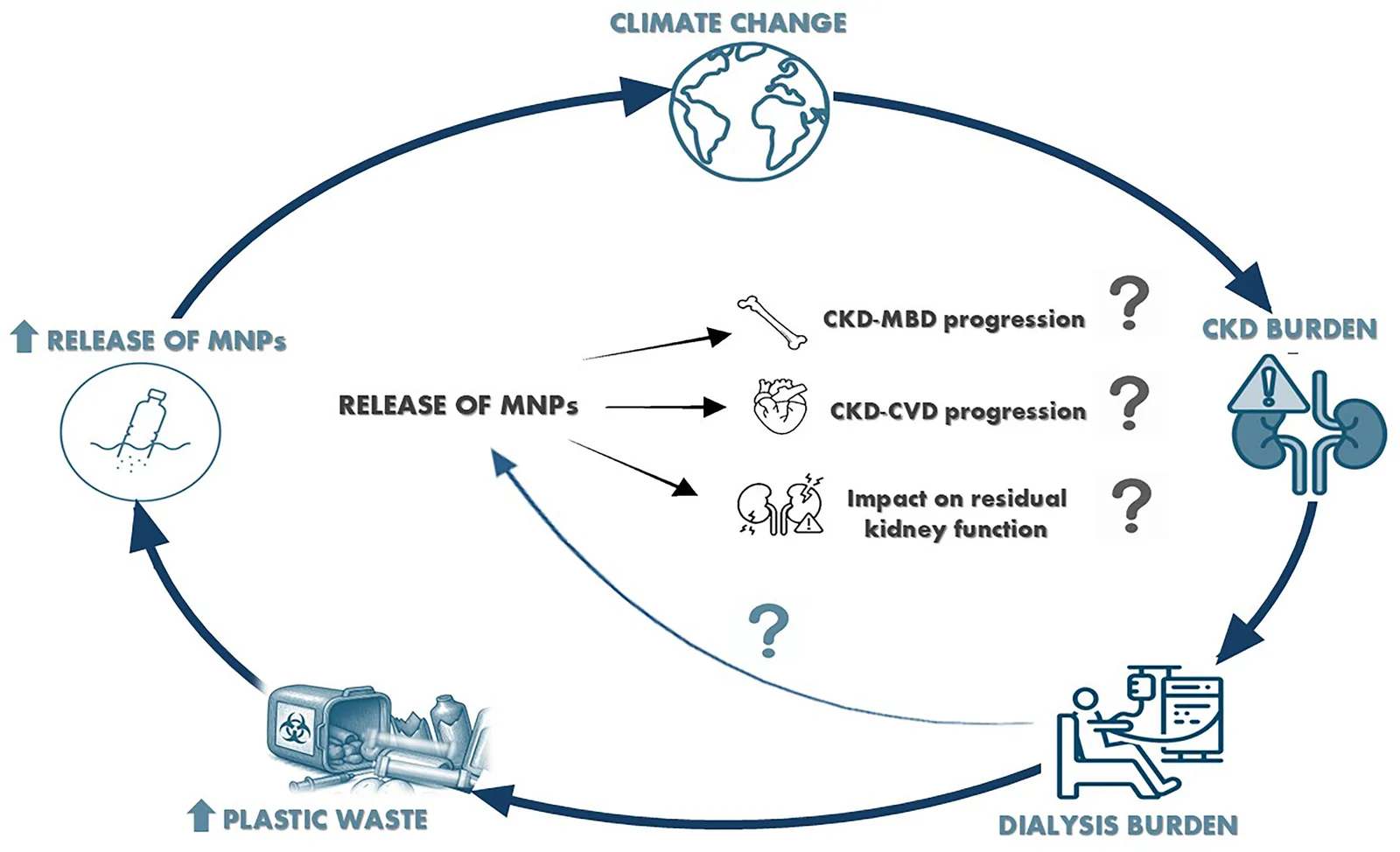
Climate change and kidney care. Climate change exacerbates the burden of CKD and, consequently, the demand for dialysis. Kidney care, particularly dialysis therapy, produces large amounts of plastic waste with significant environmental impacts, contributing to the release of MNPs and further accelerating climate change. Patients with CKD on dialysis may be exposed to substantial amounts of MNPs, which may be, theoretically, a potential driver of major cardiovascular and bone complications associated with CKD.

Abbreviations – CVD: cardiovascular disease; CKD-MBD: chronic kidney disease-mineral and bone disorder; CKD: chronic kidney disease; MNPs: microplastics and nanoplastics.

Note – Image created using the Canva platform (canva.com).

A primary example of this environmental footprint is water consumption. During a 4-hour hemodialysis (HD) session with a dialysate flow of 500 mL/min, not only is 120 L of water required, but total water consumption can reach nearly 500 L when reject water is taken into account^2,8^. This represents a critical strategic concern in HD^6,7,8^.

The amount of plastic waste generated per dialysis session represents another important environmental concern^1^. Depending upon the type of dialysis machine, supplies used, and local waste management systems, the amount of plastic waste generated per session ranges from 1.5 to 8 kg^1,2^.

The projected growth in the prevalence of patients under dialysis treatment is closely linked to the rising demand for plastic materials. This is just one example of the plastic production burden worldwide, contributing, at least partially, to the projections of the Organization for Economic Co-operation and Development: plastic use will nearly triple by 2060^23^, and its production will generate substantial CO^2^ emissions, directly contributing to climate change.

Despite this expansion, a substantial proportion of plastic waste will continue to be landfilled, with less than 20% being recycled^23^ Given the rapid increase in plastic production, plastic waste has caused widespread environmental pollution and, consequently, generated an enormous amount of MNPs through its degradation^1,23,24^.

## WHAT IS THE RELATIONSHIP BETWEEN DIALYSIS TREATMENT, EXPOSURE TO MNPS, AND CLINICAL OUTCOMES?

Although there is no robust scientific evidence that exposure to MNPs is related to the dialysis procedure and causes negative consequences, three studies suggest that dialysis patients may be at increased risk for exposure to MNPs^16,17,18^. This concern is reinforced by factors inherent to dialysis equipment, plastic-made medical devices, and water treatment infrastructure, which involve large volumes of water and numerous plastic components^16,17,18^.

The presence of MNPs in dialysis treatment was documented by Kara et al.^16^, who identified potential sources of MNPs in materials used to store fluids for peritoneal dialysis and hemodialysis (plastic-based dialysate storage solutions and peritoneal dialysis bags).

Deng et al.^18^ evaluated the impact of hemodialysis on MNP abundance in patients’ bloodstream using laser direct infrared imaging spectrometry. The authors detected that the number of particles of chlorinated polyethylene (CPE), ethylene vinyl acetate (EVA), polymethylmethacrylate (PMMA), polytetrafluoroethylene (PTFE), and polyurethane (PU) in the post-hemodialysis blood samples was significantly increased (46.11 [56.22] *vs*. 96.11 [68.32], *p* = 0.027). Of note, no trace of MNPs was found in pre-hemodialysis blood samples.

Passos et al.^17^ estimated the potential number of MNPs to which patients undergoing HD may be exposed based on the estimated concentration of MNPs in treated water (particles/L). They estimated that during HD, the number of MNPs entering patients’ bloodstream may range from 0.0021 to 3768 particles/week, based on water consumption of 300–600 L/week for patients undergoing one or two HD sessions per week, respectively.

The water treatment and distribution system for hemo­dialysis represents another concern. Kanwal et al.^25^ explored the possibility of MNPs in dialysis effluent. Although reverse osmosis (RO) appears to be highly efficient in filtering nanoparticles^17,26^, given the large volumes of water used during dialysis treatment, exposure to MNPs at the level of the dialyzer (capillary) remains possible^17^. Additionally, a substantial volume of water circulates through polymer-based components, which are continuously exposed to pressure and temperature variations, as well as repeated contact with chemical agents, conditions that may promote the degradation and release of MNPs.

Although plastic particles have been detected in human blood (including that of individuals with CKD on dialysis), lungs, guts, feces, bone, and reproductive tissues, their potential health effects remain largely unknown and unproven. Thus, the impact of MNPs on the health of dialysis patients is currently unknown, although CKD and exposure to MNPs are conditions associated with increased cardiovascular risk^13,27,28,29^.

## CHALLENGES AND OPPORTUNITIES TO REDUCE PLASTIC BURDEN IN DIALYSIS

### Ensuring plastic-free water quality for dialysis

Potentially harmful concentrations of MNPs in the human body remain unknown, and current evidence suggests that their presence in raw water is generally low^30^. The extremely small size of nanoplastics has made them particularly difficult to detect and study. However, with the development of new high-throughput imaging platforms such as stimulated Raman scattering (SRS) microscopy, hundreds of thousands of tiny plastic particles could be detected in bottled water^31^. However, patients undergoing HD are exposed to substantially larger volumes of water, increasing the risk of cumulative exposure. Of note, the sources of raw water differ among HD centers, and there are no standards or regulations for the presence and concentration of MNPs.

For example, conventional drinking water treatment plants, which typically employ processes such as coagulation/flocculation, sedimentation, ozonation, and filtration, cannot guarantee complete removal of MNPs^30,32^. These concerns have already entered regulatory discussions, and efforts toward regulation and standardization are underway^30,33^. However, significant challenges remain, particularly regarding reliable detection methodologies, the costs, and the effectiveness of monitoring strategies.

Batista et al.^34^ demonstrated that natural coagulants derived from plant or animal sources, such as *Moringa oleifera*, constitute an environmentally friendly alternative to conventional coagulants for removing polyvinyl chloride (PVC) microplastics from drinking water. In their study, this approach achieved removal efficiencies exceeding 98% for microplastic particles, suggesting a viable and sustainable strategy for mitigating microplastic contamination^34^.

Even if strategies aimed at reducing MNPs concentrations in raw water are implemented, the HD treatment process itself involves extensive use of plastic materials. Currently, there are no RO systems free of plastic components, although partial reductions may be feasible^35^.

In addition, the most commonly used piping materials in water treatment systems for HD are high-quality medical-grade plastics, such as cross-linked polyethylene (PEX), polyvinylidene fluoride (PVDF), and PVC^16,17,36^. These materials are constantly exposed to pressure and temperature fluctuations, as well as frequent contact with chemical agents, conditions that may favor the degradation and release of MNPs^17,37^. Although robust evidence regarding the use of stainless steel, specifically AISI 316L, in HD water treatment systems is limited, the literature demonstrates that this material provides enhanced protection against corrosion under certain conditions and could be used for specific piping sections within water treatment systems^16,38^.

Furthermore, the use of plastic-based dialysate storage solutions and peritoneal dialysis bags represents another potential source of exposure to MNPs^16^. Stricter manufacturing guidelines, regulatory control standards for the presence of MNPs, or even the use of alternative biocompatible materials could be explored as potential solutions.

Several biopolymers, such as polycaprolactone (PCL), polybutylene adipate terephthalate (PBAT), and polylactic acid (PLA), are being extensively studied as potential replacement materials^39^. Advancements in bioengineering are focused on harmonizing environmental sustainability with clinical safety standards so that these biopolymers can be used more frequently in medical materials in the future.

Investigations into the biological and clinical implications of exposure to MNPs and synthetic polymer-based dialyzers have not been sufficiently conducted. It will be necessary to develop reproducible and accessible methodologies to detect MNPs and identify biomarkers capable of monitoring the effects of exposure to MNPs in the human body, for example, through their presence in urine^40^. While these membranes are preferred due to advantages over cellulose-based membranes, limited information is available regarding their composition and potential generation of MNPs^41^.

Another concern is whether the reuse of dialyzers contributes to increased exposure to MNPs. Although some studies observed that reuse may be considered safe from a clinical and microbiological standpoint^42^, it is unknown whether dialyzer reuse results in greater exposure to MNPs compared with the use of a single-use dialyzer. Further research regarding this issue is necessary.

Finally, although dialysis patients may be theoretically at increased risk of exposure to MNPs, the magnitude of this exposure and the possibility of accumulation associated with negative outcomes remain largely unknown.

## CONCLUSION

Green dialysis has emerged as a pivotal strategy to mitigate the environmental impact of dialysis therapy. Green nephrology must address not only waste production but also patient exposure to plastic-derived contaminants. These materials not only contribute to environmental pollution but may also represent a source of exposure to MNPs. Although the potential health risks associated with exposure to MNPs have been increasingly reported, their clinical implications remain poorly understood in the context of CKD. Generating robust evidence on the presence and impact of MNPs in kidney care, while simultaneously developing innovative approaches to reduce plastic use and waste generation, represents a major scientific and clinical challenge for the future of sustainable nephrology.

## Data Availability

No new data were generated or analyzed in this study.

## References

[1] Piccoli GB, Nazha M, Ferraresi M, Vigotti FN, Pereno A, Barbero S (2015). Eco-dialysis: the financial and ecological costs of dialysis waste products: is a “cradle-to-cradle” model feasible for planet-friendly haemodialysis waste management?. Nephrol Dial Transplant.

[2] Gauly A, Fleck N, Kircelli F (2022). Advanced hemodialysis equipment for more eco-friendly dialysis. Int Urol Nephrol.

[3] Lim AEK, Perkins A, Agar JWM (2013). The carbon footprint of an Australian satellite haemodialysis unit. Aust Health Rev.

[4] Lou-Meda R, Pérez JB (2025). Reducing the burden of chronic kidney disease in the world. Lancet.

[5] Rafferty Q, Stafford LK, Vos T, Thomé FS, Aalruz H, Abate YH (2025). Global, regional, and national prevalence of kidney failure with replacement therapy and associated aetiologies, 1990–2023: a systematic analysis for the Global Burden of Disease Study 2023. Lancet Glob Health.

[6] Barraclough KA, Berman-Parks N, Jha V, Piccoli GB, Stigant C, Talbot B (2026). Green dialysis: environmentally sustainable care, growth, and innovation: conclusions from a Kidney Disease: Improving Global Outcomes (KDIGO) Controversies Conference. Kidney Int.

[7] Barraclough KA, Agar JWM (2020). Green nephrology. Nat Rev Nephrol.

[8] Ben Hmida M, Mechichi T, Piccoli GB, Ksibi M (2023). Water implications in dialysis therapy, threats and opportunities to reduce water consumption: a call for the planet. Kidney Int.

[9] Meng X, Zhang J, Wang W, Gonzalez-Gil G, Vrouwenvelder JS, Li Z (2022). Effects of nano- and microplastics on kidney: physicochemical properties, bioaccumulation, oxidative stress and immunoreaction. Chemosphere.

[10] Beegam S, Al-Salam S, Zaaba NE, Elzaki O, Greish YE, Ali BH (2026). Polystyrene microplastics exacerbate experimental chronic kidney disease via inflammatory and oxidative pathways involving NF-κB, ERK/p38 MAPK, and sirtuin-1. Life Sci.

[11] O’Callaghan L, Olsen M, Tajouri L, Beaver D, Hudson C, Alghafri R (2025). Plastic induced urinary tract disease and dysfunction: a scoping review. J Expo Sci Environ Epidemiol.

[12] de Oliveira RB, Pelepenko LE, Masaro DA, Lustosa GMMM, de Oliveira MC, Roza NAV (2024). Effects of microplastics on the kidneys: a narrative review. Kidney Int.

[13] Marfella R, Prattichizzo F, Sardu C, Fulgenzi G, Graciotti L, Spadoni T (2024). Microplastics and Nanoplastics in Atheromas and Cardiovascular Events. N Engl J Med.

[14] Yang Y, Zhang F, Jiang Z, Du Z, Liu S, Zhang M (2024). Microplastics are associated with elevated atherosclerotic risk and increased vascular complexity in acute coronary syndrome patients. Part Fibre Toxicol.

[15] Pelepenko LE, de Oliveira MC, Masaro DA, Lustosa GMMM, Mazon T, Castilho RF (2025). Effects of microplastics on the bones: a comprehensive review. Osteoporos Int.

[16] Kara E, Konur K, Koca YŞ, Aytan Ü (2025). Unveiling hidden contaminants: a systematic quantification and characterization of microplastics in hemodialysis and peritoneal dialysis fluids. BMC Nephrol.

[17] Passos RS, Davenport A, Busquets R, Selden C, Silva LB, Baptista JS (2023). Microplastics and nanoplastics in haemodialysis waters: emerging threats to be in our radar. Environ Toxicol Pharmacol.

[18] Deng Y, Sun T, Long Z, Luo X, Geng X, Lin Z (2026). Microplastic entry into bloodstream via hemodialysis: A dual-simulation clinical study. Environ Pollut.

[19] GBD 2023 Chronic Kidney Disease Collaborators (2025). Global, regional, and national burden of chronic kidney disease in adults, 1990-2023, and its attributable risk factors: a systematic analysis for the Global Burden of Disease Study 2023. Lancet.

[20] World Health Organization (2021). Global patient safety action plan 2021–2030 towards eliminating avoidable harm in health care [Internet].

[21] Zhang J, Wang B, Ye B, Zhu B, Liu Y (2025). Association between heatwave exposure and rapid kidney function decline: a longitudinal cohort study from CHARLS. BMC Public Health.

[22] Borg MA, Bi P (2021). The impact of climate change on kidney health. Nat Rev Nephrol.

[23] Organization for Economic Co-Operation and Development (2022). Global plastics outlook.

[24] Costa J (2025). Bridging the gap: a review on the interaction between (micro)plastics and climate change. Philos Trans A Math Phys Eng Sci.

[25] Kanwal A, Han J, Xu J, Xue X, Akhmedova N, Gao Z (2026). Microplastics in dialysis effluent: environmental chemistry insights and metal-organic frameworks-based remediation prospects. Environ Toxicol Pharmacol.

[26] Pinto PE, Giacobbo A, Almeida GM, Rodrigues MAS, Bernardes AM (2025). Pressure-driven membrane processes for removing microplastics. Membranes.

[27] Tereshchenko LG, Posnack NG (2019). Does plastic chemical exposure contribute to sudden death of patients on dialysis?. Heart Rhythm.

[28] Nguyen TD, Schulze PC (2023). Cardiac metabolism in heart failure and implications for uremic cardiomyopathy. Circ Res.

[29] Augey L, Berdougo-Tritz J, Mbarek A, Jadoul M, Massy Z, Deray G (2026). Effects of microplastics and nanoplastics on the kidneys. Nephrol Dial Transplant.

[30] Barbier JS, Dris R, Lecarpentier C, Raymond V, Delabre K, Thibert S (2022). Microplastic occurrence after conventional and nanofiltration processes at drinking water treatment plants: preliminary results. Front Water.

[31] Qian N, Gao X, Lang X, Deng H, Bratu TM, Chen Q (2024). Rapid single-particle chemical imaging of nanoplastics by SRS microscopy. Proc Natl Acad Sci USA.

[32] Keerthana Devi M, Karmegam N, Manikandan S, Subbaiya R, Song H, Kwon EE (2022). Removal of nanoplastics in water treatment processes: A review. Sci Total Environ.

[33] Pulido-Reyes G, Magherini L, Bianco C, Sethi R, von Gunten U, Kaegi R (2022). Nanoplastics removal during drinking water treatment: Laboratory- and pilot-scale experiments and modeling. J Hazard Mater.

[34] Batista GS, Ferreira VAS, Godoy LGR, Moruzzi RB, Sharifi S, Reis AG (2026). Removal of microplastics from drinking water by Moringa oleifera seed: comparative performance with alum in direct and in-line filtration systems. ACS Omega.

[35] Barrientos-Riosalido A, Exposito N, Torres EA, Emiliano P, Valero F, Nadal M (2025). Removal and reincorporation of microplastics at several stages of two seawater reverse osmosis plants that provide drinking water to Barcelona metropolitan area. Desalination.

[36] Coulliette AD, Arduino MJ (2013). Hemodialysis and water quality. Semin Dial.

[37] Świetlik J, Magnucka M (2025). Aging of drinking water transmission pipes during long-term operation as a potential source of nano- and microplastics. Int J Hyg Environ Health.

[38] Memon AM, Imran IH, Alhems LM (2023). Neural network based corrosion modeling of Stainless Steel 316L elbow using electric field mapping data. Sci Rep.

[39] Ali SS, Elsamahy T, Abdelkarim EA, Al-Tohamy R, Kornaros M, Ruiz HA (2022). Biowastes for biodegradable bioplastics production and end-of-life scenarios in circular bioeconomy and biorefinery concept. Bioresour Technol.

[40] Angeletti A, Esposito P, Verzola D, Cravedi P, La Porta E (2024). Can plastics in urine serve as a noninvasive marker of systemic accumulation?. Kidney Int.

[41] Chen YA, Ou SM, Lin CC (2022). Influence of Dialysis Membranes on Clinical Outcomes: From History to Innovation. Membranes.

[42] Ribeiro IC, Roza NAV, Duarte DA, Guadagnini D, Elias RM, de Oliveira RB (2019). Clinical and microbiological effects of dialyzers reuse in hemodialysis patients. J Bras Nefrol.

