# Supplementary material for: Microplastics and dialysis: a new challenge for green nephrology
Source: J Bras Nefrol. 2026 Nov 2;48(4):e20260094. doi: 10.1590/2175-8239-JBN-2026-0094en (PMC13739468; doi:10.1590/2175-8239-JBN-2026-0094en)
Supplement: Translation

# Translation: Microplásticos e diálise: um novo desafio para a nefrologia verde

**DOI:** 10.1590/2175-8239-JBN-2026-0094pt

**Authors:** Rodrigo Bueno de Oliveira, Eric Aragão Corrêa, Peter Stenvinkel

**Affiliations:** 1 Universidade Estadual de Campinas, Faculdade de Ciências Médicas, Departamento de Clínica Médica, Campinas, SP, Brasil.; 2 Universidade Estadual de Campinas, Faculdade de Ciências Médicas, Laboratório para o Estudo Mineral e Ósseo em Nefrologia, Campinas, SP, Brasil.; 3 Karolinska University Hospital, Department of Renal Medicine, Stockholm, Sweden.

**Keywords:** Microplásticos, Nanoplásticos, Insuficiência Renal Crônica, Diálise, Diálise Renal, Diálise Verde, Nefrologia Verde

## Abstract

A nefrologia verde emergiu como uma estratégia fundamental para mitigar os impactos ambientais relacionados ao tratamento dos rins. Nesse contexto, a redução dos resíduos plásticos e a redução da pegada de carbono estão intimamente interligadas, uma vez que ambas visam minimizar o consumo desnecessário de recursos e a carga ambiental associada à assistência à saúde. O rápido crescimento da produção e do uso de plásticos levou à disseminação da poluição ambiental e à geração de enormes quantidades de micro e nanoplásticos (MNPs) por meio da degradação de materiais plásticos. Evidências crescentes sugerem que a exposição aos MNPs pode representar riscos potenciais à saúde humana. No entanto, importantes questões permanecem sem resposta sobre a relação entre a doença renal crônica (DRC), as terapias dialíticas e a exposição aos MNPs. Em particular, a magnitude dessa exposição, o potencial de acúmulo em pacientes com função renal comprometida e as possíveis consequências clínicas ainda são amplamente desconhecidos. Neste artigo de opinião, discutimos os desafios emergentes e as potenciais oportunidades na interface entre microplásticos, diálise e nefrologia verde.

## INTRODUÇÃO

Em uma única sessão de diálise podem ser utilizados até 8 kg de materiais plásticos e centenas de litros de água^1,2,3^. Com o aumento exponencial do número de pessoas acometidas pela doença renal crônica (DRC) e a necessidade cada vez maior da terapia renal substitutiva (TRS), o impacto ambiental projetado associado ao tratamento dialítico é enorme^4,5^. Reduzir a pegada ecológica relacionada a esse tipo de tratamento é um verdadeiro desafio^6^.

Nos últimos 15 anos, estratégias têm sido discutidas para oferecer um tratamento dialítico sem agredir o planeta, por meio de uma gestão eficiente de recursos, práticas de economia circular, prevenção da DRC e de suas complicações e um engajamento tanto dos pacientes quanto da comunidade. Essas estratégias são mundialmente denominadas “nefrologia verde” (“*green nephrology*”)^1,6,7,8^.

Além dessas questões ambientais, evidências recentes sugerem a liberação de micropartículas (< 5 mm) e nanopartículas (< 1 µm) de materiais plásticos (MNPs) com efeitos biológicos deletérios em células eucarióticas e animais mamíferos, incluindo seres humanos^9,10,11^.

Embora os efeitos dos MNPs na patogênese de doenças ainda sejam amplamente desconhecidos, dados na literatura relatam a presença de MNPs em praticamente todos os órgãos do corpo humano^12,13,14,15^. Sabe-se atualmente que os MNPs podem se acumular no tecido renal e ser excretados na urina, e estudos experimentais sugerem que eles podem induzir efeitos biológicos adversos nos rins^10,11,12^.

É plausível especular que, com a progressão da doença renal, os pacientes possam apresentar uma redução na capacidade de eliminar MNPs pela urina, embora ainda não exista confirmação científica sobre esse aspecto. Apesar de limitadas, evidências atuais sugerem que pacientes com DRC podem estar expostos a diversas fontes de plásticos. Dois estudos detectaram MNPs em fluidos de hemodiálise e diálise peritoneal; outro estudo baseado em modelagem matemática sugere que a hemodiálise pode liberar quantidades significativas de MNPs diretamente na corrente sanguínea^16,17,18^.

Neste artigo de perspectiva/opinião, discutiremos as evidências disponíveis sobre MNPs e o tratamento dialítico, apresentando nossa visão integrada ao contexto da *green nephrology*. Paralelamente, abordaremos as preocupações, os desafios e as oportunidades que envolvem o tratamento dialítico dentro dos princípios da *green nephrology*, com atenção especial a este novo contaminante ambiental: os MNPs.

## COMO A DRC E A CARGA DA DIÁLISE ESTÃO RELACIONADAS COM AS MUDANÇAS CLIMÁTICAS?

A DRC acomete 14,2% da população adulta mundial, tornando-se uma das doenças não transmissíveis (DNTs) mais prevalentes e de crescimento mais rápido do mundo^19^. Existem 788 milhões de pessoas acometidas pela doença renal no mundo; 3,6 milhões estão em algum tipo de TRS, e 1,5 milhão de pessoas morrem anualmente com DRC. Globalmente, a prevalência da DRC varia substancialmente entre regiões e populações específicas, com estimativas variando de cerca de 5 a 20%, dependendo do país e de fatores de risco como diabetes, hipertensão e condições socioeconômicas. Por exemplo, taxas muito mais altas são registradas no Norte da África, Oriente Médio, Sul da Ásia e América Central em comparação com regiões de alta renda *per capita* ^4^. Essa tendência alarmante contribuiu para a decisão da Organização Mundial da Saúde (OMS) de reconhecer a DRC como uma DNT de prioridade global crescente^4,20^.

O aumento das temperaturas médias globais, decorrente das mudanças climáticas, tem o potencial de agravar a carga ligada à DRC, uma vez que está associado a riscos significativos para a saúde renal^21,22^. Em uma época de avanço das mudanças climáticas e disrupção ecológica, os rins podem servir como os “canários na mina de carvão”, ou seja, sentinelas da saúde pública: órgãos altamente sensíveis e biologicamente responsivos que registram sobrecarga hemodinâmica, osmótica, inflamatória e tóxica muito antes que padrões mais amplos de doenças se manifestem plenamente.

Este ciclo se fecha, já que os cuidados com a saúde renal em portadores de DRC exigem recursos ambientais sub­stanciais e geram grandes quantidades de resíduos plásticos e, consequentemente, MNPs, contribuindo para um impacto ambiental (Figura 1)^1,3,8^.

**Figura 1:** Mudanças climáticas e saúde dos rins. As mudanças climáticas agem como um fator agravante na carga da DRC e, consequentemente, na demanda por diálise. O tratamento renal, especialmente a terapia dialítica, gera grandes quantidades de resíduos plásticos com impactos ambientais significativos, contribuindo para a liberação de MNPs e acelerando ainda mais as mudanças climáticas. Pacientes com DRC em diálise podem ser expostos a quantidades substanciais de MNPs, o que pode ser, teoricamente, um possível fator para complicações clínicas maiores da DRC relacionadas à doença cardiovascular e aos ossos.

Abreviaturas – DCV: doença cardiovascular; DMO; distúrbio mineral e ósseo; DRC; doença renal crônica; MNPs; microplásticos e nanoplásticos.

Notas – Imagem criada utilizando a plataforma Canva (canva.com).

Um exemplo claro dessa questão ambiental é o consumo de água. Durante uma sessão de hemodiálise (HD) de 4 horas com fluxo de dialisato de 500 mL/min, não apenas são necessários 120 L de água, mas o consumo total de água pode chegar a quase 500 L quando a água de rejeito é levada em consideração^2,8^. Isso representa uma preocupação estratégica em relação à HD^6,7,8^.

A quantidade de resíduos plásticos gerada em cada sessão de diálise representa outra preocupação ambiental importante^1^. Dependendo do tipo de máquina de diálise, dos insumos utilizados e dos sistemas locais de gestão de resíduos, a quantidade de lixo plástico gerada por sessão pode variar de 1,5 a 8 kg^1,2^.

O crescimento projetado da prevalência de pacientes em diálise está intimamente ligado ao aumento da demanda por materiais plásticos. Este é apenas um exemplo do fardo da produção de plástico em todo o mundo, o qual contribui, ao menos parcialmente, para as projeções da Organização para a Cooperação e Desenvolvimento Econômico: o uso de plástico quase triplicará até 2060^23^, e sua produção gerará emissões substanciais de CO^2^, contribuindo diretamente para as mudanças climáticas.

Apesar dessa expansão, uma proporção substancial dos resíduos plásticos continuará a ser enviada para aterros sanitários, com menos de 20% sendo reciclados^23^. Dado o rápido crescimento da produção, o lixo plástico tem causado uma poluição ambiental generalizada e, consequentemente, gerado uma enorme quantidade de MNPs por meio de sua degradação^1,23,24^.

## QUAL A RELAÇÃO ENTRE TRATAMENTO DIALÍTICO, EXPOSIÇÃO AOS MNPS E DESFECHOS CLÍNICOS?

Embora não haja evidências científicas robustas de que a exposição aos MNPs esteja relacionada com o tratamento dialítico e cause consequências negativas, três estudos sugerem que pacientes em diálise podem apresentar um risco aumentado de exposição a esses materiais^16,17,18^. Essa preocupação é reforçada por fatores inerentes aos equipamentos de diálise, aos dispositivos médicos fabricados com materiais plásticos e à infraestrutura de tratamento de água, os quais envolvem grandes volumes e inúmeros componentes plásticos^16,17,18^.

A presença de MNPs no tratamento dialítico foi documentada por Kara et al.^16^, que identificaram potenciais fontes desses materiais em insumos utilizados para o armazenamento de fluidos para diálise peritoneal e hemodiálise (soluções de armazenamento de dialisato em material plástico e bolsas de diálise peritoneal).

O estudo de Deng et al.^18^ avaliou, por meio de espec­trometria de imagem por infravermelho direto a laser (LDIR), o impacto da hemodiálise na concentração de MNPs na corrente sanguínea dos pacientes. Os autores detectaram que o número de partículas de MNPs de polietileno clorado (CPE), etileno-vinil-acetato (EVA), polimetilmetacrilato (PMMA), politetrafluoretileno (PTFE) e poliuretano (PU) nas amostras de sangue pós-hemodiálise aumentou significativamente (46,11 [56,22] *vs*. 96,11 [68,32]; *p* = 0,027). Notavelmente, nenhum traço de MNPs foi encontrado nas amostras de sangue antes do início da sessão de hemodiálise.

Passos et al.^17^ estimaram a quantidade de MNPs aos quais os pacientes em HD podem estar expostos com base na concentração estimada de MNPs na água tratada (partículas/L). Segundo as estimativas, durante a HD, o número de MNPs que entram na corrente sanguínea dos pacientes pode variar de 0,0021 a 3.768 partículas/semana, com base em um consumo de água de 300 a 600 L/semana para pacientes submetidos a uma ou duas sessões de HD por semana, respectivamente.

O sistema de tratamento e distribuição de água para hemodiálise também representa uma preocupação. Esse aspecto foi explorado no artigo de revisão de Kanwal et al.^25^, no qual os autores investigaram a possibilidade de presença de MNPs no efluente da diálise. Embora a osmose reversa (OR) pareça altamente eficiente na filtragem de nanopartículas^17,26^, estimativas sugerem que, dados os grandes volumes de água utilizados durante o tratamento dialítico, a exposição aos MNPs no nível do dialisador permanece possível^17^. Adicione a isso o fato de que um volume substancial da água para hemodiálise circula por componentes à base de polímeros, os quais são continuamente expostos a variações de pressão e temperatura, bem como ao contato repetido com agentes químicos, condições que podem favorecer a degradação e a liberação de MNPs.

Portanto, embora partículas de plástico tenham sido detectadas no sangue (incluindo indivíduos com DRC em diálise), pulmões, intestinos, fezes, ossos e tecidos reprodutivos humanos, seus potenciais efeitos sobre a saúde, incluindo aqueles em indivíduos com DRC, permanecem em grande parte desconhecidos e não comprovados. Assim, apesar de o impacto da exposição aos MNPs sobre a saúde dos pacientes em diálise ainda ser desconhecido, tanto a DRC quanto a exposição aos MNPs estão associadas a um maior risco cardiovascular^13,27,28,29^.

## DESAFIOS E OPORTUNIDADES PARA REDUZIR A CARGA DE PLÁSTICO RELACIONADA À DIÁLISE

### Garantindo a qualidade da água livre de plásticos para a diálise

Concentrações potencialmente prejudiciais de MNPs no corpo humano permanecem desconhecidas e as evidências atuais sugerem que sua presença na água bruta é, de modo geral, baixa^30^. O tamanho extremamente reduzido dos nanoplásticos torna sua detecção e estudo particularmente difíceis. Com o avanço no desenvolvimento de novas plataformas de imageamento de alto rendimento, como a microscopia por espalhamento Raman estimulado (SRS), centenas de milhares de minúsculas partículas de plástico puderam ser detectadas em água engarrafada^31^. No entanto, os pacientes submetidos à HD são expostos a volumes substancialmente maiores de água, o que pode potencialmente aumentar o risco de exposição cumulativa. Vale ressaltar que as fontes de água bruta variam entre os centros de HD e não existem padrões ou regulamentações para a presença e concentração de MNPs.

Por exemplo, as estações convencionais de tratamento de água potável, que normalmente empregam processos como coagulação/floculação, sedimentação, ozonização e filtração, não podem garantir a remoção completa de MNPs^30,32^. Essas preocupações já foram incorporadas às discussões regulatórias, e esforços para estabelecer regulamentações e padronizações estão em andamento^30,33^. Porém, desafios significativos ainda persistem, particularmente no que diz respeito ao desenvolvimento de metodologias confiáveis de detecção, bem como aos custos e à eficácia das estratégias de monitoramento.

Batista et al.^34^ demonstraram que coagulantes naturais derivados de fontes vegetais ou animais, como *Moringa oleifera*, constituem uma alternativa ambientalmente sustentável aos coagulantes convencionais para a remoção de microplásticos de policloreto de vinila (PVC) da água potável. Neste estudo, essa abordagem alcançou eficiências de remoção superiores a 98% para partículas de microplásticos, sugerindo uma estratégia viável e sustentável para a mitigação da contaminação por microplásticos^34^.

Mesmo que sejam implementadas estratégias destinadas a reduzir as concentrações de MNPs na água bruta, o próprio processo de tratamento por HD envolve o uso extensivo de materiais plásticos. Atualmente, não existem sistemas de OR completamente livres de componentes plásticos, embora seja possível reduzir parcialmente a utilização desses componentes^35^.

Os materiais de tubulação mais comumente utilizados nos sistemas de tratamento de água para HD são plásticos de alta qualidade, como o polietileno reticulado (PEX), o fluoreto de polivinilideno (PVDF) e o policloreto de vinila (PVC)^16,17,36^. Estes materiais estão constantemente expostos a variações de pressão e temperatura, bem como ao contato frequente com agentes químicos, condições que podem favorecer sua degradação e a liberação de MNPs^17,37^. Embora evidências robustas sobre o uso de aço inoxidável, especificamente o AISI 316L, em sistemas de tratamento de água para HD sejam limitadas, a literatura demonstra que esse material oferece maior proteção contra a corrosão em determinadas condições e poderia ser utilizado em trechos específicos das tubulações dos sistemas de tratamento de água^16,38^.

O uso de soluções à base de plástico para o armazenamento do dialisato e de bolsas utilizadas na diálise peritoneal representa outra fonte potencial de exposição aos MNPs^16^. Diretrizes de fabricação mais rigorosas, padrões de controle regulatório para a presença de MNPs ou mesmo o uso de materiais biocompatíveis alternativos poderiam ser explorados como possíveis soluções.

Diversos biopolímeros, como a policaprolactona (PCL), o adipato-tereftalato de polibutileno (PBAT) e o ácido polilático (PLA), vêm sendo amplamente estudados como potenciais materiais substitutos^39^. Os avanços na bioengenharia têm se concentrado em conciliar a sustentabilidade ambiental com os padrões de segurança clínica, de modo que esses biopolímeros possam ser utilizados com maior frequência em materiais médicos no futuro.

Até o momento, não foram realizadas investigações sobre as implicações biológicas e clínicas da exposição aos MNPs em pacientes submetidos à hemodiálise com dialisadores à base de polímeros sintéticos. Nesse sentido, será necessário desenvolver metodologias reprodutíveis e acessíveis para a detecção de MNPs, bem como identificar biomarcadores capazes de monitorar os efeitos desta exposição no organismo humano, por exemplo, por meio de sua detecção na urina^40^. Embora essas membranas sejam preferidas devido às suas vantagens em relação às membranas à base de celulose, ainda existem informações limitadas sobre sua composição e seu potencial de geração de MNPs^41^.

Outra preocupação diz respeito à possibilidade de o reuso dos dialisadores contribuir para o aumento da exposição aos MNPs. Embora alguns estudos tenham observado que sua realização é considerada segura do ponto de vista clínico e microbiológico^42^, ainda não se sabe se resultaria em uma maior exposição aos MNPs em comparação com o uso de dialisadores de uso único. São necessárias pesquisas adicionais sobre essa questão.

Por fim, embora os pacientes em diálise possam, teorica­mente, apresentar maior risco de exposição aos MNPs, a magnitude dessa exposição e a possibilidade de acúmulo associado a desfechos adversos permanecem, em grande parte, desconhecidas.

## CONCLUSÃO

A diálise verde (g*reen dialysis*) vem se consolidando como uma estratégia fundamental para mitigar o impacto ambiental da terapia dialítica. A nefrologia verde (g*reen nephrology*) deve abordar não apenas a geração de resíduos, mas também a exposição dos pacientes a contaminantes derivados de plásticos. Esses materiais não apenas contribuem para a poluição ambiental, mas também podem representar uma fonte de exposição aos MNPs. Embora os potenciais riscos à saúde associados a esta exposição tenham sido cada vez mais relatados, suas implicações clínicas ainda são pouco compreendidas. A geração de evidências robustas sobre a presença e o impacto dos MNPs no tratamento dialítico, aliada ao desenvolvimento de abordagens inovadoras para reduzir o uso de plásticos e a geração de resíduos, representa um importante desafio científico e clínico para o futuro de uma nefrologia sustentável.

## Data Availability

Nenhum dado novo foi gerado ou analisado nesse estudo.
